# Response to: ‘Acute stroke requires sophisticated and demanding management to achieve an optimal outcome’

**DOI:** 10.51866/lte.1110

**Published:** 2026-04-21

**Authors:** Afiza Hanun Ahmad@Hamid, Nor Shahrina Mohd Zawawi, Mohd Fairuz Ali

**Affiliations:** 1 Klinik Kesihatan Sitiawan, Sitiawan, Perak, Malaysia.; 2 Department of Medical Rehabilitation Services, Hospital Canselor Tuanku Muhriz (HCTM), National University of Malaysia (UKM), Kuala Lumpur, Malaysia.; 3 Department of Family Medicine, Medical Faculty, Hospital Canselor Tuanku Muhriz (HCTM), National University of Malaysia (UKM), Kuala Lumpur, Malaysia.

**Keywords:** Cognitive training, Return to work, Rehabilitation

**Dear Editor,**

We are grateful for the authors’ interest regarding our published report entitled ‘Primary care management of mild cognitive impairment in a stroke survivor: A case report on facilitating return to work’.^1^ We welcome this opportunity to clarify the concerns they raised in the letter to editor and to furnish additional information to the readers.^2^

## Pre-stroke cognitive status

We acknowledge the importance of assessing baseline cognitive function. At presentation, there was no history suggestive of cognitive impairment, based on information obtained from the patient and corroborated through interviews with his wife. We agree that the absence of formal pre-stroke cognitive assessment represents a limitation and recognise that a structured collateral history would be valuable in similar situations in the future.

## Vascular risk factor control

The patient’s cardiovascular profile prior to the stroke event was unavailable. At the time of stroke diagnosis, his HbAlc level was 6.5%, indicating good diabetes control. His lipid profile showed an elevated LDL cholesterol level (3.5 mmol/L) with a total cholesterol level of 5.4 mmol/L. These details were not included in the brief case summary due to word limitations. Furthermore, 24-hour ambulatory blood pressure monitoring was not conducted during the acute phase. We acknowledge that more comprehensive monitoring and documentation of these metabolic and vascular parameters prior to the stroke would have provided a more complete assessment of his risk profile.

## Cardiac evaluation

The patient had no history of angina; his ECG showed normal sinus rhythm, and there were no findings suggestive of recent or past myocardial infarction. Thus, coronary angiography was not routinely conducted according to the Malaysian guideline.^3^ We concur that a more extensive cardiac work-up may be warranted for young patients presenting with cerebrovascular events.

## Cognitive testing

The Montreal Cognitive Assessment, a validated screening tool,^4^ was used to check for mild cognitive impairment. The use of a comprehensive neuropsychological battery for detailed cognitive profiling was not possible because of limited resources and difficulties with patient follow-up. The patient was nevertheless referred for further cognitive evaluation to support return-to-work planning. An occupational therapist administered the Rivermead Perceptual Assessment (RPA) to aid in the patient’s rehabilitation, although further cognitive testing was still required for return-to-work planning. Due to extended waiting times, the RPA was not completed until 4 months after the stroke. The patient achieved acceptable scores across all domains, demonstrating good focus, concentration, perceptual abilities and judgement. We recognise the need for more timely and thorough cognitive evaluation and intend to improve this process in the future.

## Terminology of motor deficits

We appreciate the clarification regarding the term ‘haemiplegic gait’. The patient had mild left lower limb weakness and required assistance from his wife for ambulation. His gait was uneven, with slower and less controlled movement of the affected limb, causing him to rely on the contralateral limb for support. We agree that the term ‘haemiparetic gait’ is more appropriate and regret the error.

## Post-stroke depression

We agree that post-stroke depression is common and may confound the assessment of cognitive function.^5^ In this case, the patient was screened using the Patient Health Questionnaire-9, which did not indicate clinical depression. However, we acknowledge the limitations of relying on a single screening instrument and recognise the need for continued evaluation, particularly when interpreting cognitive symptoms in the post-stroke context.

## Use of ‘recent stroke’

The patient first presented to a general practitioner approximately 4 hours after the onset of haemiparesis and was advised immediate hospital referral. However, he presented to the hospital 24 hours later, by which time symptoms were mild, and he was discharged. One week later, he presented to a health clinic with a second episode of left-sided weakness, prompting an urgent computed tomography scan of the brain. Imaging demonstrated ill-defined hypodense areas in the right temporal lobe, corona radiata, basal ganglia and occipital lobe, with preserved basal cisterns and no midline shift or intracranial haemorrhage. Based on the clinical history and imaging findings, the changes were interpreted as consistent with a recent stroke. He was referred to a specialist clinic but defaulted follow-up and therefore did not undergo further investigations or receive thrombolysis or thrombectomy.

## Imaging

In our setting, magnetic resonance imaging (MRI) was performed in selected cases due to resource limitations.^3^ Access to MRI in Malaysian primary care is restricted and requires hospital physician or neurologist referral. Although expedited physician review was arranged in this case, the patient did not attend, and MRI was not performed. With an improvement in cognitive symptoms and return to work, he declined further medical referral and continued follow-up at the health clinic.

## Follow-up imaging

Follow-up imaging was not conducted during cognitive rehabilitation, since there were no new focal deficits or clinical features. We believe serial imaging would allow for better differentiation of post-stroke cognitive impairment and recurrent events and will apply this in future practice and clinical protocols.

## Conclusion

We appreciate the constructive feedback and acknowledge the limitations highlighted. We hope that our response contributes meaningfully to ongoing discussions regarding cognitive sequelae following stroke. These are the challenges faced at the primary care level, particularly limited access to assessment resources. Nevertheless, ongoing efforts are being made to strengthen management at the primary care level through co-management with hospital-based expertise to enhance the quality and continuity of stroke rehabilitation services in Malaysia.
